# Scalable parameterized quantum circuits classifier

**DOI:** 10.1038/s41598-024-66394-2

**Published:** 2024-07-10

**Authors:** Xiaodong Ding, Zhihui Song, Jinchen Xu, Yifan Hou, Tian Yang, Zheng Shan

**Affiliations:** Laboratory for Advanced Computing and Intelligence Engineering, Zhengzhou, 450001 China

**Keywords:** Quantum information, Quantum simulation, Computer science

## Abstract

As a generalized quantum machine learning model, parameterized quantum circuits (PQC) have been found to perform poorly in terms of classification accuracy and model scalability for multi-category classification tasks. To address this issue, we propose a scalable parameterized quantum circuits classifier (SPQCC), which performs per-channel PQC and combines the measurements as the output of the trainable parameters of the classifier. By minimizing the cross-entropy loss through optimizing the trainable parameters of PQC, SPQCC leads to a fast convergence of the classifier. The parallel execution of identical PQCs on different quantum machines with the same structure and scale reduces the complexity of classifier design. Classification simulations performed on the MNIST Dataset show that the accuracy of our proposed classifier far exceeds that of other quantum classification algorithms, achieving the state-of-the-art simulation result and surpassing/reaching classical classifiers with a considerable number of trainable parameters. Our classifier demonstrates excellent scalability and classification performance.

## Introduction

With the development of quantum computing technology^1^, quantum machine learning^2–15^ has become a hot research field, and multi-category classification is one of the important tasks. Conventional multi-category classification algorithms are typically based on deep learning frameworks^16–18^, but these methods require large amounts of data and computational resource, and suffer from issues such as overfitting. Quantum machine learning, which combines quantum computing and machine learning, has the advantages of accelerating computation and reducing overfitting, making it widely applicable to various problems. However, for classification problems, most existing quantum machine learnings focus on binary classification tasks, and for multi-category classification problems, the research mainly focuses on deploying classical neural networks at the end of quantum algorithms^19^. Quantum machine learnings based on measurement of projection^9^, QF-hNet^2^ and other methods^20–22^ have also been proposed for solving the multi-category classification problem, but the classification effect and scalability are poor. It is worth looking forward to providing quantum multi-category classifiers with good scalability and excellent classification results. Therefore, in this paper, we propose SPQCC with PQC^23,24^ as the core. This choice is made because PQC, as a generalized quantum machine learning model for quantum machine learning, has the properties of certain resilience to certain types of errors, coherence time, and more flexible operation attributed to the properties of quantum parallelism, superposition and entanglement, which has shown strong learning capability and is now used as a core module for algorithms such as QNN^25–27^, QCNN^28,29^, QLSTM^30,31^, and QGAN^32,33^. The number of parallel PQCs of this classifier is the same as the number of classes of samples, which has good scalability. Secondly, the multiple PQCs have the same structure and scale, and only needs to design one channel of PQC to complete the design of parallel multiple PQCs of the classifier, which makes the design of the classifier more convenient. Meanwhile, these PQCs are allowed to be executed in parallel, so the execution efficiency of the classifier is higher. Finally, the measurements of the parallel multiple PQCs are combined as the output of the final classifier, and the trainable parameters of all the PQCs are optimized by minimizing the cross-entropy loss function, which leads to fast convergence of the classifier. Additionally, in this paper we also emphasize on the design of PQC, the circuit measurement methods and parameters optimization.

## Results

**Table 1:** As far as the classification problem of the dataset is concerned, Total sample count, Training sample count and Test sample count vary and the table shows the detailed information for different datasets.

Problem	Total sample count	Training sample count	Test sample count
2-class 1,5	13298	12163	2027
2-class 3,6	14017	12049	1968
2-class 3,8	13966	11982	1984
2-class 3,9	14099	12080	2019
3-class 0,3,6	20920	17972	2948
3-class 1,3,6	21894	18791	3103
4-class 0,3,6,9	27878	23921	3957
5-class 0,1,3,6,9	35755	30663	5092
5-class 0,1,2,3,4	35735	30596	5139
10-class 0,1,2,3,4,5,6,7,8,9	70000	60000	10000

**Figure 1:**
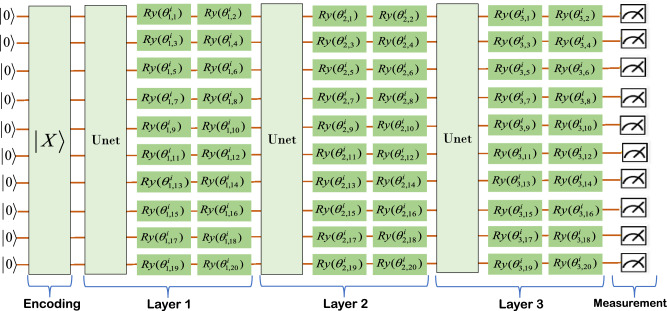
One channel PQC Design for Classifier over MNIST dataset, where ${\vec {\theta } ^i}$ in the PQC could denote the weight matrix *W* in the traditional neural networks, PQC in the figure could be expressed by the following equation:$W({\vec {\theta } ^i}) = {U_{net}}{U_l}(\vec {\theta } _3^i){U_{net}}{U_l}(\vec {\theta } _2^i){U_{net}}{U_l}(\vec {\theta } _1^i)$, ${U_{net}} = \prod \limits _{(i,j) \in E} {CZ(i,j)}$, ${U_l}(\vec {\theta } _j^i) = \otimes _{k = 1}^{20,2}{R_y}(\vec {\theta } _{j,k}^i){R_y}(\vec {\theta } _{j,k + 1}^i)$, $\otimes _{k = 1}^{20,2}$ denote *k* from 1 to 20, increasing by 2 each time.

**Figure 2:**
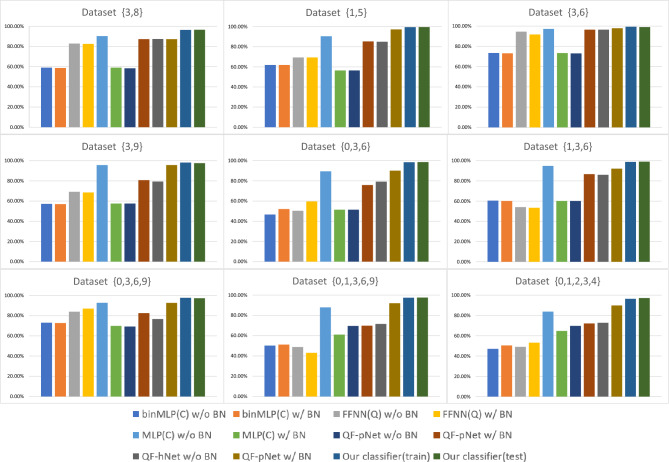
TBinMLP(C) w/o BN, BinMLP(C) w/ BN, FFNN(Q) w/o BN, FFNN(Q) w/ BN,, MLP(C) w/o BN, MLP(C) w/ BN, QF-pNet w/o BN, QF-pNet w/ BN, QF-hNet w/o BN, QF-pNet w/ BN, Our classifier(train), Our classifier(test) Different algorithms on MNIST sub-datasets $\left\{ {1,5} \right\} ,\left\{ {3,6} \right\} ,\left\{ {3,8} \right\} ,\left\{ {3,9} \right\} ,\left\{ {0,3,6} \right\} ,\left\{ {1,3,6} \right\} ,\left\{ {0,3,6,9} \right\} ,\left\{ {0,1,3,6,9} \right\} ,\left\{ {0,1,2,3,4} \right\}$ Classification Accuracy Display Graph.

The scalability and classification effectiveness of our proposed classifier are verified. The scalability of the model is primarily demonstrated in two aspects: the ability to handle datasets with varying numbers of categories and the ability to adjust the model’s size. Scalability when dealing with datasets with different numbers of categories: When faced with datasets containing varying numbers of categories, the model must be capable of adapting flexibly while maintaining good performance. Specifically, the model can be scaled by simply adjusting the number of PQCs in the model based on the number of categories in the dataset. This approach ensures that the model is both robust and flexible when dealing with datasets containing varying numbers of categories. Scalability regarding the size of the model: This involves not only an expansion in the number of PQCs, but also the adjustment of the number of layers in the quantum circuits. We chose the MNIST dataset for separate classification simulations on the quantum simulator TensorCircuit. This dataset was chosen because for more than a decade, researchers from the fields of Machine Learning, Machine Vision, Artificial Intelligence, and Deep Learning have used this dataset as one of the benchmarks for measuring classification algorithms^34–38^. We compare the classification accurate of our classifier with the classification accurate of other classifiers^9,39^ on the MNIST dataset. To carry out the experimental validation, we have equipped the following hardware facilities: Processor: We used an Intel Core i7-8700K, a powerful processor with strong computing power and multi-threaded processing ability, to meet the experimental needs. Memory: The computer is equipped with 64GB of DDR4 RAM, ensuring sufficient memory resources to keep the experiment running efficiently when processing large amounts of data.In this experiment, we used the following hyperparameter configurations: Learning Rate: We chose 0.01 or the default 0.001 as the learning rate, depending on the specific dataset.Batch Size (Batch Size): We used 64 as the batch size. Iteration Count (Epochs): We set the iteration count to 50.Optimizer: We chose the Adam optimizer. Loss Function: We chose the CategoricalCrossentropy function. We perform data preprocessing by resize the input images from $28\times 28$ to $32\times 32$, equivalent to the usage of 10-qubits system on the quantum hardware. In order to better demonstrate the advantages of our classifier. This experiment was used to validate the model’s ability to handle datasets with varying numbers of categories. We perform 2,3,4,5 classification on the sub-datasets $\left\{ {1,5} \right\} ,\left\{ {3,6} \right\} ,\left\{ {3,8} \right\} ,\left\{ {3,9} \right\} ,\left\{ {0,3,6} \right\} ,\left\{ {1,3,6} \right\} ,\left\{ {0,3,6,9} \right\} ,\left\{ {0,1,3,6,9} \right\} ,\left\{ {0,1,2,3,4} \right\}$ to compare the performance of our method on the training and testing datasets. Total Sample Count, Total Sample Count, and Total Sample Count vary depending on the classification problem of the dataset. Each dataset contains a specific classification problem and is trained and tested using the corresponding samples. These details are crucial for understanding the structure of the dataset, evaluating model performance, and comparing different approaches. We perform model evaluation on different classes of classification problems, and the details of the evaluation dataset are provided in Table 1. Set relative to BinMLP(C) w/o BN, BinMLP(C) w/BN, FFNN(Q) w/o BN, FFNN(Q) w/ BN, MLP(C) w/o BN, MLP(C) w/ BN, QF-pNet w/o BN, QF-pNet w/ BN, QF-hNet w/o BN, QF-pNet w/ BN and other algorithms for classification accuracy. For MNIST actual data features, we design PQC as shown in Fig. 1. For multi-category classification, our classifier only needs to replicate the circuit in Fig. 1 by configuring different trainable parameters for the corresponding number of times, without any additional procedures required. This approach can be extended to support *N* classes, indicating good scalability of our classifier. For the MNIST sub-datasets $\left\{ {1,5} \right\} ,\left\{ {3,6} \right\} ,\left\{ {3,8} \right\} ,\left\{ {3,9} \right\} ,\left\{ {0,3,6} \right\} ,\left\{ {1,3,6} \right\} ,\left\{ {0,3,6,9} \right\} ,\left\{ {0,1,3,6,9} \right\} ,\left\{ {0,1,2,3,4} \right\}$ the classification accurate on the different algorithms are shown in Fig. 2. The validation results in Fig. 2 clearly indicate that the classification accuracy of our proposed classifier on various subsets of the MNIST dataset, both for training and testing, is noticeably better than that of other quantum classification approaches, establishing it as the current state-of-the-art simulation result and surpassing (or matching) classical classifiers (e.g., MLPs) with a significant number of trainable parameters.

**Figure 3:**
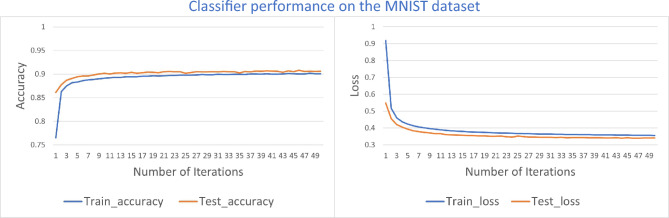
Through 50 epochs of training and testing, the classification accuracy and loss function variation of classifier. Classifier achieves 90% classification accuracy on both the training and testing Datasets. Classifier has good convergence by converging to the optimal model quickly after 20 epochs.

To further validate the scalability and classification accuracy of our classifier, we extend the sub-dataset to the entire MNIST dataset. For our proposed classifier, it only requires parallelizing ten channels to complete the ten-category classification of the entire dataset. At the same time, we adjust the number of layers of the parameterized quantum circuit from three to four, thereby completing the experimental verification. This experiment is used to verify the ability of the model to adjust the size of the scale. After 50 epochs of training and testing, the classification accuracy and loss function evolution of the classifier are plotted in Fig. 3.

On the MNIST dataset, our classifier achieved a ten-category classification accuracy, where 50 epochs of iterative training were performed. For each epoch, the model was tested on the testing dataset, and the rate of change of classification accuracy and loss value on both the training and testing datasets throughout the iterations is plotted in Fig. 3. Our proposed classifier achieved a classification accuracy of 90% on both the training and testing datasets, and the classification accuracy of projection valued measure-based quantum machine learning for multi-category classification^9^ was less than 80%. Our classifier was 10% higher in classification accuracy and showed good convergence by fast converging to the optimal model after 20 epochs. We have made the source code of all our experiments publicly available through the GitHub platform (https://github.com/zhaoding3/xiaodong/), aiming to enable the general public to directly access, review, and validate the core aspects of our experimental environment configurations, data processing flow, and model training, thus ensuring the transparency of our research work and the reproducibility.

## Discussion

Multi-category classification is a crucial task in the field of machine learning. Conventional multi-category classification methods require significant amounts of data and computational resources, and suffer from issues such as overfitting. Existing quantum machine learnings mostly focus on binary classification problems, and the research on multi-category classification problems has poor classification accuracy and scalability. Therefore, SPQCC has better scalability while ensuring classifier performance. Our classifier requires PQCs to have the same structure and scale, making it possible for the algorithm designer to design only one channel of PQC to complete the design of the whole classifier, greatly reducing the complexity of the design. Moreover, after designing a one-channel PQC, only the number of parallel PQCs equals to the classification class can be naturally extended to multi-category classifications, from which we could see that our proposed classifiers are more scalable. Additionally, our classifier realizes parallel execution of PQCs regardless of the number of classification classes, and the training time used is only related to the sample scale and the result and scale of one channel of PQC, but not the number of channels of PQCs executed, which has the same efficiency as that of the same scale of parameterized quantum circuit-based quantum machine learning algorithms. Our model employs the method of multiple PQCs. With each additional parameterized quantum circuit or increase in the number of layers of PQCs, the number of parameters in the whole model increases significantly, although the increase in the depth of the quantum circuit is not significant. Deeper circuits need to be designed to account for this additional parameter compared to the traditional single circuit model. However, designing deeper circuits poses a number of challenges. For instance, the coherence time of quantum bits, deeper circuit design may also introduce more errors and noise. This extended approach ensures the performance and stability of the model when dealing with more complex and larger datasets. Finally, our classifier is compared with existing classifiers in terms of classification effectiveness, and the experimental results illustrate that our proposed classifier exhibits excellent classification accuracy.

## Methods

### Classifier framework

**Figure 4:**
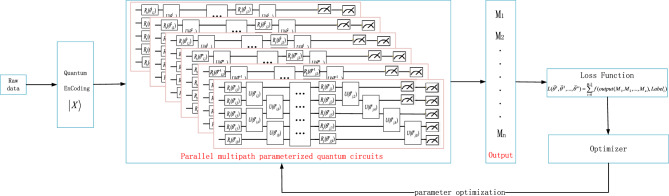
The SPQCC framework consists of four main components: quantum encoding, parallel multi-channel PQCs, quantum circuits measurement, and loss function minimization for parameter optimization.

Our proposed SPQCC belongs to a variant of quantum neural networks, which has four main components: quantum encoding, parallel multi-channel PQCs, quantum circuits measurement, and loss function minimization for parameters optimization. Its model is shown in Fig. 4.

Quantum encoding: Similar to other quantum machine learning algorithms, the implementation of SPQCC first requires mapping vectors to quantum states in the Hilbert space using features, which is generally achieved by quantum encoding for this process. The main encoding methods^40^ at this stage are base encoding, amplitude encoding, repetitive amplitude encoding, rotational encoding, coherent state encoding, and so on. Amplitude encoding, approximate amplitude encoding, and rotational encoding are commonly used to map features from classical data to quantum states. Quantum amplitude coding is effective for certain problems, but implementing it can require a large number of quantum gate operations, posing computational complexity and scalability challenges. Approximate amplitude encoding^41,42^, as an important encoding method, is implemented by training shallow PQCs to encode given classical data into quantum circuits. Compared with quantum amplitude encoding, it uses fewer gates and shallower circuit depths. However, this encoding approach requires multiple training sessions to encode classical data, and thus also suffers from certain encoding efficiency issues. Rotational encoding is usually easier to understand and implement than other quantum encoding methods, and they are better resistant to noise and interference, making them more advantageous in quantum communication and quantum computing. But rotational encoding requires more quantum resources to accomplish computational tasks, potentially making them less practical in resource-limited systems. The choice of encoding method to implement feature mapping usually depends on factors such as the model designer’s experience, the characteristics of the original data, the number of bits in the quantum computer, and the decoherence time of the quantum system^43^. In order to meet the requirements of the validation experiment, we chose amplitude coding to realize feature mapping based on the characteristics of the experimental dataset and hardware conditions. In this paper, we focus on feature mapping using amplitude encoding, the core idea of which is to utilize the properties of quantum interference and quantum entanglement to encode vectors into the amplitude of a quantum state, which is processed in the form of a qubit, which has an exponential advantage in terms of memory. Amplitude encoding requires that the vectors are first normalized before encoding: ${x_{ij}} = {x_{ij}}/\left\| {{{\vec {x}}_i}} \right\|$, which has a general form: $f({\vec {x}_i}) = \sum \nolimits _{j = 1}^n {{x_{ij}}} \left| j \right\rangle$. The main way to implement amplitude encoding is the iterative approach, where the basic process is that the encoding of new quantum states is accomplished by multiple control operations of the already encoded generated quantum states on those that need to be encoded until all the features of the vector are encoded.

Parallel Multi-channel PQCs: Parallel multi-channel PQCs serve as the core component of the classifier, each of which features the same structure and scale. The heart of PQC is built from trainable quantum gates containing parameters, which in turn constitutes modules of the unit layer through these parameter-bearing quantum gates. Based on actual needs, the modules of the unit layer are stacked to create PQC. For different application scenarios and problems, PQC of different structures and scales need to be designed according to the specific circumstances.

Quantum Circuit Measurement: Measurement is performed on each qubit in a quantum circuit^44,45^. In real quantum computers, the measurement is usually done through multiple iterations and the final results are tallied, which are presented in the form of vectors composed of quantum states and corresponding probabilities. Generally, to verify the correctness of the results of the method, quantum simulators could be leveraged, such as PennyLane^46^, Qiskit^47^, and TensorCircuit^48^.

Parameters Optimization: Similarly to quantum neural networks, SPQCC requires the definition of a loss function^49^ to quantify the difference between the predicted and true values. Here, we choose the cross-entropy^50^ loss function, and during training, the parameters in the classifier are iteratively updated by a gradient descent algorithm. In PQC models, the gradients of the computed parameters are typically estimated using traditional automatic differentiation methods, although they could also be calculated using the parameter-shift rule and gate decomposition^51^ of quantum circuits.

In what follows, we will focus on the design of PQC, the quantum circuit measurement, loss function selection, and parameters optimization.

### Parallel multi-channel PQCs

The PQCs in our proposed classifier all have the same structure and scale, making it possible to design only one channel PQC to meet the requirements of the classifier. This concept greatly simplifies and accelerates the design process of the classifier. The core of PQC is the trainable parameters contained in the Ansatz quantum gates, and the quantum gates containing trainable parameters in this paper are mainly composed of basic quantum gates of the form ${e^{ - i\theta G/2}}(G = \{ X,Y\} )$ and two-qubit gates $U = {e^{i\theta (Y \otimes Y)}}$,${U_1} = {e^{i\theta (Z \otimes Z)}}$, where

1 $$\begin{aligned} {R_x}(\theta )= & {} {e^{ - i\theta X/2}} = \cos \frac{\theta }{2}I - i\sin \frac{\theta }{2}X = \left[ {\begin{array}{*{20}{l}} {\cos \frac{\theta }{2}}&{}\quad {-i\sin \frac{\theta }{2}}\\ {-i\sin \frac{\theta }{2}}&{}\quad {\cos \frac{\theta }{2}} \end{array}} \right] \end{aligned}$$

2 $$\begin{aligned} {R_y}(\theta )= & {} {e^{ - i\theta Y/2}} = \cos \frac{\theta }{2}I - i\sin \frac{\theta }{2}Y = \left[ {\begin{array}{*{20}{l}} {\cos \frac{\theta }{2}}&{}\quad { - \sin \frac{\theta }{2}}\\ {\sin \frac{\theta }{2}}&{}\quad {\cos \frac{\theta }{2}} \end{array}} \right] \end{aligned}$$

**Figure 5:**
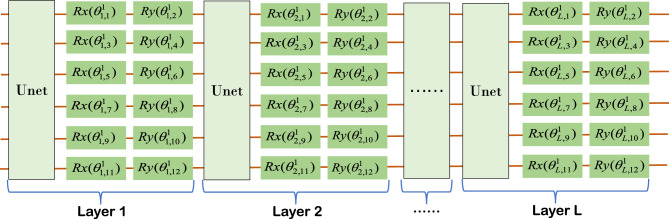
The diagram of the first PQC, where ${\vec {\theta } ^1}$ in the PQC could denote the weight matrix *W* in the traditional neural networks, PQC in the figure could be expressed by the following equation:$W({\vec {\theta } ^1}) = {U_{net}}{U_l}(\vec {\theta } _L^1){U_{net}}{U_l}(\vec {\theta } _{L - 1}^1)...{U_{net}}{U_l}(\vec {\theta } _2^1){U_{net}}{U_l}(\vec {\theta } _1^1)$ (*L* is the number of layers), ${U_{net}} = \prod \limits _{(i,j) \in E} {CZ(i,j)}$, ${U_l}(\vec {\theta } _j^1) = \otimes _{k = 1}^{n,2}{R_x}(\vec {\theta } _{j,k}^1){R_y}(\vec {\theta } _{j,k + 1}^1)$, $\otimes _{k = 1}^{n,2}$ denote *k* from 1 to *n*, increasing by 2 each time, and *n* is the number of parameters in each layer.

**Figure 6:**
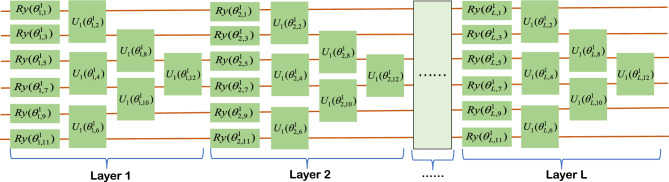
The diagram of the first PQC, where ${\vec {\theta } ^1}$ in the PQC could denote the weight matrix *W* in the traditional neural networks, PQC in the figure could be expressed by the following equation:$W({\vec {\theta } ^1}) = \otimes _{j = 1}^L{U_l}(\vec {\theta } _j^1)$ (*L* is the number of layers), ${U_l}(\vec {\theta } _j^1) = \otimes _{k = 1}^{n,2}{R_y}(\vec {\theta } _{j,k}^1){U_1}(\vec {\theta } _{j,k + 1}^1)$, $\otimes _{k = 1}^{n,2}$ denote *k* from 1 to *n*, increasing by 2 each time, and *n* is the number of parameters in each layer.

**Figure 7:**
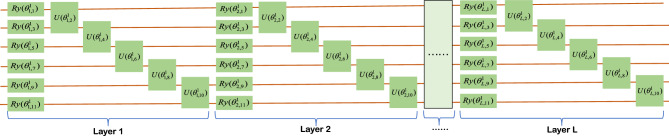
The diagram of the first PQC, where ${\vec {\theta } ^1}$ in the PQC could denote the weight matrix *W* in the traditional neural networks, PQC in the figure could be expressed by the following equation:$W({\vec {\theta } ^1}) = \otimes _{j = 1}^L{U_l}(\vec {\theta } _j^1)$ (*L* is the number of layers), ${U_l}(\vec {\theta } _j^1) = \otimes _{k = 1}^{n,2}{R_y}(\vec {\theta } _{j,k}^1){U_1}(\vec {\theta } _{j,k + 1}^1)$, $\otimes _{k = 1}^{n,2}$ denote *k* from 1 to *n*, increasing by 2 each time, and *n* is the number of parameters in each layer.

The matrices $X,Y,Y \otimes Y,Z \otimes Z$ are obviously unitary matrices, which meet the requirement that the operations of a quantum system must be unitary matrices. PQC contains only ${R_x}(\theta )$ and ${R_y}(\theta )$. The first step to do is to apply a *CNOT* gate between each pair of qubits to ensure that it generates quantum entanglement between qubits in Hilbert space, and the basic structure of the qubits in terms of six for example is shown in the Fig. 5. By including ${U}(\theta )$ and ${U_1}(\theta )$ in the basic layer, topological PQCs can realize quantum state entanglement without the need to apply *CNOT* gates between each pair of qubits. Illustrated here as an example using six qubits, two different topological basic structures are shown: the block structure in Fig. 6 and the ladder structure in Fig. 7. The trainable parameters $\vec {\theta }$ in PQC are analogous to the adjustable weights *W* in conventional neural networks^52^, and the loss functions are constructed by measuring the expectation values of various observations on the PQCs. In PQCs, we initialize the parameters $\vec {\theta } = ({\vec {\theta } ^1},{\vec {\theta } ^2},....,{\vec {\theta } ^n})$, where *n* denotes the number of categories and ${\vec {\theta } ^j}$ represents the parameters of the *j*th channel of the PQC. In SPQCC ${\vec {\theta } ^1},{\vec {\theta } ^2},....,{\vec {\theta } ^n}$ are treated as a whole, and in the process of parameters optimization, $\vec {\theta } = ({\vec {\theta } ^1},{\vec {\theta } ^2},....,{\vec {\theta } ^n})$ need to be updated for each channel for each optimization step. In summary, the design of PQC for a classifier allows us to determine the structure and scale of PQC according to the needs of different application scenarios, and then select appropriate quantum gates from the parameter-containing quantum gates to complete the design. This process is full of possibilities, and the diversity of PQCs designed are also an attraction of our classifier. Finally, assuming that the number of categories is *m*, the design of the classifier can be completed by parallelizing the *m* designed PQC.

### Quantum circuit measurement

Our measurement is performed on PQC where the qubit state is measured in a standard basis (Z-basis)^53,54^. The Z-basis is the basis used to measure whether a qubit is in the $\left| 0 \right\rangle$ state or the $\left| 1 \right\rangle$ state, which is determined by the state of the wavefunction of the qubit prior to the measurement. The process of performing a measurement is to project a qubit from the superposition state onto the standard basis states $\left| 0 \right\rangle$ and $\left| 1 \right\rangle$, and the measurement causes the state of the qubit to undergo a collapse to a fixed standard basis state $\left| 0 \right\rangle$ or $\left| 1 \right\rangle$, we record the probability of the state collapsing to $\left| 0 \right\rangle$, and then sum the probabilities of all the qubits collapsing to $\left| 0 \right\rangle$ of each qubit of PQC as output, corresponding to the n-category classification, and the whole outputs of the classifier results in ${M_1},{M_2},...,{M_n}$.

The specific measurement is, for the measurement under the Z-basis, the two corresponding measurement operators are ${Z_0} = \left| 0 \right\rangle \left\langle 0 \right| ,{Z_1} = \left| 1 \right\rangle \left\langle 1 \right|$, which would be seen to be self-adjoint, i.e., ${Z_0}^\dag = {Z_0},{Z_1}^\dag = {Z_1}$, and satisfy ${Z_0}^2 = {Z_0},{Z_1}^2 = {Z_1}$. Let the state of a qubit when it is measured be $\left| \varphi \right\rangle = \alpha \left| 0 \right\rangle + \beta \left| 1 \right\rangle$ , and the state in which the measurement result is 0 be $p(0) = \left\langle \varphi \right| {Z_0}^\dag {Z_0}\left| \varphi \right\rangle = \left\langle \varphi \right| {Z_0}\left| \varphi \right\rangle = {\left| \alpha \right| ^2}$. Let one-channel PQC contain *m* qubits, then according to our quantum circuit measurement method, the output result of each quantum circuit is: ${M_i} = \sum \nolimits _{j = 0}^m {{p_j}(0)}$.

### Loss function design and parameters optimization

SPQCC, like quantum neural networks, requires the definition of loss function to measure the difference between the predicted value and the true value. As mentioned in parallel PQCs, the qubits of each PQC are measured at each qubit on *Z*. That is, each qubit is projected onto the ground state separately, and the probability of collapsing to $\left| 0 \right\rangle$ is calculated as ${P_i}$. After adding all the ${P_i}s$ of the PQC together and the sum ${M_i}$ as the output, the outputs of the whole classifier are ${M_1},{M_2},...,{M_n}$. After combining the results and passing them through the $\text {SoftMax}$^55,56^ as the final outputs.$\text {SoftMax}$ mainly transforms the output value of the multi-category classification into a probability distribution in the range of [0,1] with sum 1. At the same time we use one-hot^57,58^ to encode the sample labels, which provides the basis for generating the loss function for parameters optimization. $Soft\max ({M_i}) = \frac{{{e^{{M_i}}}}}{{\sum \nolimits _{c = 1}^C {{e^{{M_c}}}} }}$, ${M_i}$ is the output of the i-th PQC, and *C* is the number of parallel PQCs (the number of classes to be classified). For the loss function we choose the cross-entropy, and the $\text {SoftMax}$ maps the output of the classifier ${M_1},{M_2},...,{M_n}$ to a vector ${\hat{y}}$, ${\hat{y}} = Soft\max ({M_1},{M_2},...,{M_n})$, which we could consider as the estimated conditional probability of each class for an arbitrary sample *x*.

Let assume that the dataset $\{ X,Y\}$ has *n* samples, where the samples indexed as *i* are composed of a feature vector ${x_i}$ and a corresponding vector ${y_i}$ of one-hot label. Then for any *x* corresponding to the true label *y* and the result ${\hat{y}}$ predicted by the classifier, we define the cross-entropy loss function as $l(y,{\hat{y}}) = - \sum \nolimits _{i = 1}^n {{y_i}\log {{{\hat{y}}}_i}}$. We use the one-hot labels for encoding, so in the vector *y*, only one component is 1 and the rest are 0. We could then write the loss function as: $l(y,{\hat{y}}) = - \sum \nolimits _{i = 1}^n {{y_i}\log {{{\hat{y}}}_i}}$. According to the definition of $\text {SoftMax}$, the loss function $l(y,{\hat{y}}) = - \sum \nolimits _{i = 1}^n {{y_i}\log {{{\hat{y}}}_i}}$ could be expanded as:

3 $$\begin{aligned} l(y,{\hat{y}}) = - \sum \limits _{i = 1}^n {{y_i}\log \frac{{{e^{{o_i}}}}}{{\sum \nolimits _{c = 1}^n {{e^{{o_c}}}} }}} = \sum \limits _{i = 1}^n {\log \sum \limits _{c = 1}^n {{e^{{o_c}}}} - \sum \limits _{i = 1}^n {{y_i}{o_i}} } = \log \sum \limits _{c = 1}^n {{e^{{o_c}}}} - \sum \limits _{i = 1}^n {{y_i}{o_i}} \end{aligned}$$

The loss function is derived for any prediction ${o_i}$:

4 $$\begin{aligned} \frac{{\partial l(y,{\hat{y}})}}{{\partial {o_i}}} = \frac{{e^{o_i}}}{{\sum \nolimits _{c = 1}^n {{e^{{o_c}}}} }} - {y_i} = Soft\max {(o)_i} - {y_i} \end{aligned}$$

From the above equation, the derivative is the difference between the output obtained by the multi-category classifier and the true value.

The parameters in the classifier are updated iteratively during training through a gradient descent algorithm. The gradient of the computed parameters for PQC is typically estimated using the traditional automatic differentiation method, or the parameter-shift rule of the quantum circuit can be applied. The PQC, with the phase in the quantum gate serving as the primary training parameters, causes the initial quantum state $\left| {{\varphi _{in}}} \right\rangle$ to evolve into the desired quantum final state $\left| {{\varphi _{out}}} \right\rangle$ through iterative training, i.e., $\left| {{\varphi _{out}}} \right\rangle = W(\vec {\theta } )\left| {{\varphi _{in}}} \right\rangle$ , where $W(\vec {\theta } )$ represents the corresponding quantum state of the PQC. The optimization of the parameters focuses on reducing the deviation between the predicted and true values, quantified by the loss function $l(y,{\hat{y}}) = - \sum \nolimits _{i = 1}^n {{y_i}\log {{{\hat{y}}}_i}}$, where ${{\hat{y}}_i}$ and ${y_i}$ represent the predicted and true values corresponding to ${x_i}$, respectively. Through continuous training, the loss function is minimized, or the error of its loss function is brought within an acceptable range. For the simulations in this paper, we utilize the traditional automatic differentiation method to compute the gradient solutions.

Frontier research^1,59–61^ has made it clear that quantum neural networks, through the superposition and entanglement properties of qubits, have demonstrated significant advantages compared to classical neural networks exhibit significant advantages, including fewer parameters, lower resource requirements, faster training, and lower risk of overfitting, which enable efficient representation of complex functional relationships. Our model incorporates the core advantages of quantum neural networks. In addition, our model adopts a multiplexed PQC architecture, assuming that each channel contains *m* parameters and consists of *c* channels, resulting in a total number of $m*c$ parameters. In terms of the use of qubits, the number of qubits required for amplitude encoding is $c*\big \lceil {\log n} \big \rceil$ if a parallel structure is used, and only $\big \lceil {\log n} \big \rceil$ qubits are required if a no-parallel structure is used, where *n* is the data dimension. In this case, the required resources are exponentially reduced compared to classical neural networks.

## Conclusion

PQC is one of the mainstream models of quantum machine learning, which mainly stacks a set of quantum gates containing parameters together to form a model. Optimization of these parameters through training is necessary to achieve the desired output. Multi-category classification has numerous applications and is a worthwhile research problem. SPQCC, which we propose, takes advantage of the parallelism of PQCs, merges the measurements of the model as the final output of the classifier, and minimizes the cross-entropy loss function for optimizing the classifier’s parameters. This method allows for establishing the same number of PQCs corresponding to the number of classes according to the number of classes, using the same measurement method. The PQCs need only be designed once in the design process of the classifier, with the same design complexity as designing a single PQC. Additionally, the time complexity is equivalent to that of a single PQC classifier using parallel processing and multiple simulators. However, our designed classifier fully utilizes the advantages of quantum computing and has better scalability. We tested it on MNIST, and the classification accuracy is similar to that of traditional methods. Our findings provide new ideas and methods for solving multi-category classification problems using PQC, and contribute to the performance and efficiency of quantum algorithms for solving multi-category classification problems. In future work, we will build on this foundation to achieve multi-category classification using quantum perceptron machines.

## Data Availability

All data generated or analysed during this study are included in this published article.
